# Evidence for an Allosteric *S*-Nitrosoglutathione Binding Site in *S*-Nitrosoglutathione Reductase (GSNOR)

**DOI:** 10.3390/antiox8110545

**Published:** 2019-11-13

**Authors:** Kathleen Fontana, Nneamaka Onukwue, Bei-Lei Sun, Cristina Lento, Leslie Ventimiglia, Sahar Nikoo, James W. Gauld, Derek J. Wilson, Bulent Mutus

**Affiliations:** 1 Department of Chemistry and Biochemistry, University of Windsor, 401 Sunset Ave., Windsor, ON N9B 3P4, Canada; katie.fontana94@gmail.com (K.F.); onukwue@uwindsor.ca (N.O.); ventimi@uwindsor.ca (L.V.); nikoo@uwindsor.ca (S.N.); gauld@uwindsor.ca (J.W.G.); 2 Apotex Inc., 150 Signet Dr., North York, ON N9B 3P4, Canada; bei.sun127@gmail.com; 3 Department of Chemistry, York University, 4700 Keele Street, Toronto, ON N9B 3P4, Canada; clento@yorku.ca (C.L.); dkwilson@yorku.ca (D.J.W.)

**Keywords:** *S*-nitrosoglutathione reductase, GSNOR, hydrogen–deuterium exchange mass spectroscopy, allosteric site for *S*-nitrosoglutathione, GSNO, docking and molecular dynamics simulations

## Abstract

Current research has identified *S*-nitrosoglutathione reductase (GSNOR) as the central enzyme for regulating protein *S*-nitrosylation. In addition, the dysregulation of GSNOR expression is implicated in several organ system pathologies including respiratory, cardiovascular, hematologic, and neurologic, making GSNOR a primary target for pharmacological intervention. This study demonstrates the kinetic activation of GSNOR by its substrate *S*-nitrosoglutathione (GSNO). GSNOR kinetic analysis data resulted in nonhyperbolic behavior that was successfully accommodated by the Hill–Langmuir equation with a Hill coefficient of +1.75, indicating that the substrate, GSNO, was acting as a positive allosteric affector. Docking and molecular dynamics simulations were used to predict the location of the GSNO allosteric domain comprising the residues Asn185, Lys188, Gly321, and Lys323 in the vicinity of the structural Zn^2+^-binding site. GSNO binding to Lys188, Gly321, and Lys323 was further supported by hydrogen–deuterium exchange mass spectroscopy (HDXMS), as deuterium exchange significantly decreased at these residues in the presence of GSNO. The site-directed mutagenesis of Lys188Ala and Lys323Ala resulted in the loss of allosteric behavior. Ultimately, this work unambiguously demonstrates that GSNO at large concentrations activates GSNOR by binding to an allosteric site comprised of the residues Asn185, Lys188, Gly321, and Lys323. The identification of an allosteric GSNO-binding domain on GSNOR is significant, as it provides a platform for pharmacological intervention to modulate the activity of this essential enzyme.

## 1. Introduction

*S*-nitrosoglutathione (GSNO) is formed in response to nitric oxide (NO) fluxes by the reaction of oxidized forms of NO (e.g., N_2_O_3_) with the free thiol of glutathione. GSNO can in turn modulate protein *S*-nitrosylation via transnitrosylation—that is, the transfer of a NO^+^ moiety to protein thiols. A large body of evidence indicates that the under or over *S*-nitrosylation of proteins has a great impact on many pathologies [1]. 

*S*-nitrosoglutathione reductase (GSNOR), coded by the alcohol dehydrogenase ADH5 gene in humans, was first identified by Koivusalo et al. [2] as a NAD^+^-dependent aldehyde dehydrogenase. In 1998, Jensen et al. [3] reported that ADH5 could also catalyze the NADH-coupled reduction of *S*-nitrosoglutathione (GSNO) to N-hydroxysulphenamido glutathione (Equation (1)). In the presence of glutathione (GSH), N-hydroxysulphenamido glutathione is converted to hydroxylamine and glutathione disulfide (GSSG) (Equation (2)).

GSNOR GSNO + NADH → GSNHOH + NAD^+^ (1)

non enzymatic GSNHOH + GSH → NH_2_OH + GSSG (2)

Additional studies indicated that ADH5 and its GSNO metabolic activity are conserved from prokaryotes to eukaryotes [4]. ADH5, which is expressed in all human tissues [5,6], was renamed GSNOR by researchers in the *S*-nitrosothiol (SNO) signaling field.

GSNO metabolic activity is not specific to GSNOR. Super oxide dismutase [7], glutathione peroxidase [8], protein disulfide isomerase [9], thioredoxin [10], and carbonyl reductase [11] can all metabolize GSNO. However, only GSNOR and carbonyl reductase can reduce NO^+^ to NH_2_OH, which cannot be converted back to NO_x_ via biological systems. In addition, the GSNO metabolic activity of only thioredoxin and GSNOR have been demonstrated to be physiologically relevant [12]. 

GSNOR (ADH5) is generally found to be elevated in respiratory, cardiovascular, smooth muscle, and autoimmune disease states. Many studies indicate that the lowering of GSNOR (AHD5) has beneficial effects. For example, the deletion of GSNOR promotes bronchodilation and protection against asthma in the lungs [13], improves post-cardiac arrest resuscitation [14], increases cell senescence in mouse and human models [15], protects against autoimmune disease [16], improves skeletal muscle resistance and fatigue resistance [17], and increases cardiomyocyte proliferation [18] and neuronal differentiation [19]. 

Interest in GSNOR as a therapeutic target is growing. Several drugs that inhibit GSNOR were developed for treating asthma, cystic fibrosis, and chronic obstructive pulmonary disease (COPD) [14,15,16]. More recently, the demonstration of a direct role for GSNOR in dysfunctional relaxation in preterm labor has sparked interest in GSNOR inhibitors as potential new tocolytic drugs.

Clearly, controlling the activity of GSNOR is of ongoing pharmacological interest. The current study could potentially provide this opportunity, as it not only presents evidence for an allosteric GSNO binding site, but it also shows that the activity of GSNOR is very sensitive to perturbations at this site.

## 2. Materials and Methods

### 2.1. Materials

All reagents were purchased from Sigma-Aldrich (Oakville, ON, Canada) unless otherwise stated in the methods section.

All solutions were prepared with MilliQ water (Advantage A10 Water Purification System, Millipore Sigma, Etobicoke, ON, Canada).

### 2.2. Methods

#### 2.2.1. GSNOR WT Cloning, Mutagenesis, and Protein Isolation

##### GSNOR (ADH5) Sub-Cloning

Human GSNOR (ADH5) was purchased from Origene (SC119755) and subcloned into bacterial expression vector pET28b using Cold Fusion Cloning Kit, SYMC010A1 (MJS BioLynx Inc., Brockville, ON, Canada). The destination vector pET28b was linearized via digestion with restriction enzymes NdeI and XhoI, PCR amplified and purified by electrophoresis on agarose gels.

The primers used for GSNOR (ADH5-ScSFA1) subcloning were: 

Forward 5′– GTGCCGCGCGGCAGCCATATGGCGAACGAGGTTATCAAG –3′

Reverse 5′– GTGGTGGTGGTGGTGCTCGAGAATCTTTACAACAGTTCGAATG –3′.

The ligated plasmid DNA was transformed directly into chemically competent *E. coli*. Colonies were screened using diagnostic restriction enzyme digest and by partial sequencing (Robarts Research, London, ON, Canada). 

##### GSNOR Site-Directed Mutagenesis

Site-directed mutagenesis of the residues at the postulated GSNO binding site was outsourced to Genscript. The following primers (NP_000662) for PCR were designed and sent to Genscript:

Lys188Ala Forward 5′ ACT GCC GCG TTG GAG CCT 3′
Lys188Ala Reverse 5′ GTT CAC AGC AGC ACC ATA ACC GGT 3′
Lys323Ala Forward 5′ GCC TTT GGA GGA TGG GCG AGT GTA GAA AGT GTC 3′
Lys323Ala Reverse 5′ AGT GCC TTT CCA TGT GCG ACC 3′

The resultant PCR product was treated with a kinase, ligase, and Dpn1 enzyme mix according to the manufacturer’s protocol, and used to transform chemically competent DH5 *E. coli* (New England BioLabs C2987, Whitby, ON, Canada). Individual colonies from the transformation plates were selected, and each colony was inoculated into 3 mL of LB medium (with 50 µg/mL kanamycin) for overnight growth at 37 °C. Then, plasmid DNA was isolated from these cultures using a standard bacterial miniprep procedure (Qiagen, Montreal, PQ, Canada).

#### 2.2.2. GSNOR Expression and Purification

A single transformed colony was inoculated into 25 mL of 2× YT medium containing 50 µg/mL kanamycin and cultured overnight at 37 °C with shaking. The starter culture was poured into 1 L of 2× YT medium containing 50 µg/mL kanamycin and incubated at 37 °C until an OD of approximately 0.6 was attained at which time GSNOR (*wt* and mutant) expression was induced by the addition of Image result for IPTG.

Isopropyl β-d-1-thiogalactopyranoside (IPTG) (0.4 mM). The induced culture was grown for an additional 24 h at room temperature with shaking, and the cells were harvested by centrifugation (4000× *g*, 30 min, 4 °C). The bacterial cell pellet was resuspended in lysis buffer (50 mM Tris-HCl pH 8, 150 mM NaCl, 15 mM imidazole, 1 mM dithiothreitol (DTT), 1 mM phenylmethylsulfonyl fluoride (PMSF), 0.5% Triton X-100, 50 µg/mL DNase I, and 100 µg/mL lysozyme). The suspension was incubated on ice for 30 min and pulse sonicated (30 cycles of 20 s on/20 s off) (Fisher Scientific Model FB120, Ottawa, ON, Canada). Then, the lysate was centrifuged (11,000× *g*, 30 min, 4 °C), and recombinant GSNOR in the supernatant was purified by HIS-Select^®^ Nickel Affinity Gel chromatography (Sigma-Aldrich). The buffers employed were: (i) wash buffer, 50 mM Tris-HCl pH 8, 150 mM NaCl, and 50 mM imidazole; (ii) elution buffer, 50 mM Tris-HCl pH 8, 150 mM NaCl, and 300 mM imidazole. The eluted protein was buffer exchanged into storage solution (58 mM Na_2_HPO_4_, 17 mM NaH_2_PO_4_, 68 mM NaCl, 15% glycerol) using an Amicon centrifugal filter (Millipore Sigma), divided into 100-µL aliquots, and frozen at −80 °C.

##### GSNOR Kinetics

*S*-nitrosoglutathione (GSNO) was synthesized according to the method of Hart [17]. The concentrations of GSNO and NADH were determined from the absorbance of their respective solutions using their extinction coefficients: GSNO, 922 M^−1^ cm^−1^ at 335 nm; NADH, 6220 M^−1^ cm^−1^ at 340 nm.

The kinetic studies were performed under steady-state conditions where constant concentrations of the enzyme (approximately 20 nM) and the cofactor [NADH] (80 μM) were used with varying [GSNO] (2 μM to 200 μM). The reaction was initiated by the addition of GSNOR. Due to the partial overlap of the absorption maxima of NADH and GSNO, the decrease at 340 nm due to GSNOR activity is 85% from NADH oxidation and 14% from GSNO reduction across the entire substrate range employed. The absorbance at 340 nm was monitored (Agilent 8453 UV/Vis Spectrophotometer, Mississauga, ON, Canada) for 180 s, and initial rates were extracted from the kinetic data and fitted to the Hill–Langmuir equation [18,19] (Equation (3)) where *n* is the Hill coefficient. 

The kinetic studies were performed as eight replicate experiments in triplicate.

(3) $$v_{0}=\frac{V_{max}{\left[S\right]}^{n}}{K_{M}^{n}+{\left[S\right]}^{n}}$$

### 2.3. Computational Methods

#### Docking and Molecular Dynamics (MD) Simulation

The Molecular Operating Environment (MOE) program was used to prepare all systems for docking and molecular dynamics (MD) simulations [20]. For the chemical models, an experimental X-ray crystal structure of suitably high resolution (1.9 Å) of *S*-nitrosoglutathione reductase (GSNOR) with a bound reversible inhibitor (PDB ID: 3QJ5) [21] was used as the initial template structure. The bound inhibitor was deleted from the structure. The protonation states of all residues were assigned using the Propka tool as available in the MOE program. All crystallographic waters were removed. Subsequently, the substrate *S*-nitrosoglutathione (GSNO) was docked in the whole protein using the virtual screening formulism. To evaluate the top poses, the London dG scoring function was used with the AMBER12:EHT force field refinement keeping the top 10 scores [20]. Then, molecular dynamics simulations were performed on the top scoring structure (i.e., predicted most preferred bound complex) obtained. The MD simulations were run using the NAMD program using its default settings as provided through MOE [22]. Each system was resolvated to a water depth of 6 Å away from any residue. The solvated structure generated was optimized using the AMBER12: EHT molecular mechanics force field until the root-mean-square gradient fell below 0.01 kcal/mol Å^−1^. In order to allow for thermal relaxation and equilibration, the minimized structures were each annealed over 100 ps, during which the temperature was raised from 0 to 300 k at constant pressure. Then, this was followed for each equilibrated complex by a 3-ns production run MD simulation, in which all the atoms were free to move, with a time step of 2 fs under constant pressure and temperature. It is noted that the default settings include use of the Particle mesh Ewald (PME) method for calculating Coulombic interactions, a cut-off for nonbonded interactions of 8–10 Å, and tether ranges of 0 to 100 Å applied to heavy atoms. For each production simulation, cluster analyses were performed on the conformations obtained (for instance, based on the root-mean-square deviation (rmsd) of the protein backbone, and the bound GSNO and putative binding site residues) and a representative structure was selected from the most populated cluster for further analysis.

### 2.4. MS-MS for GSNOR Peptide Identification

Tandem mass spectrometry (MS-MS) was performed on a Waters, Synapt G1 for characterization of the peptides fragmented from the pepsin column. By acquiring data in MS-TOF mode, a precursor ion can be chosen to undergo further fragmentation by collision-induced dissociation (CID) The collision energy applied was customized for each precursor ion to obtain an ideal fragmentation pattern. The fingerprint spectra were collected within a mass range of 100–2000 *m/z*.

A theoretical pepsin digest was performed using the FindPept tool on the ExPASy Proteomics server (Swiss Institute of Bioinformatics, Basel, Switzerland). Search parameters were set to pepsin (porcine A) at pH > 2 with a mass tolerance of ± 0.5 Da. The possible identities of the parent ion were chosen from the resulting list. Each possible parent ion was theoretically fragmented by the spectra-viewing software mMass and compared with the fingerprint. The parameters used included searching for the loss of –H_2_O and –NH_3_, as well as identifying y, a, b, int-a, and int-b fragment ions.

### 2.5. HDX-MS

Hydrogen–deuterium (D) exchange (HDX) MS was made possible by outfitting the Synapt G1 with the custom TRESI apparatus as described by Rob et al. [23]. The reagents utilized include 5% (*v/v*) acetic acid, GSNOR in 200 mM ammonium acetate, and deuterium oxide (D_2_O) (99.9% purity of LC-MS grade). These reagents are pumped through a polyamide-coated glass capillary with an outer diameter (o.d.) of 109.2 μm using Harvard Apparatus Pump 11 Elite infusion syringe pumps (Holliston, MA, USA). Protein and D_2_O were pumped at a rate of 2 μL/min with 0.5-mL syringes (Hamilton 700 78 Series Gastight Syringe Cole-Parmer Montreal, PQ, Canada), while acid was pumped at a rate of 16 μL/min with a 5-mL or 2.5-mL syringe (Hamilton 1000 Series Gastight Syringe). 

The 109.2-μm o.d. glass capillary-containing protein is encased in a metal capillary with an inner diameter (i.d.) of 132.6 μm. A 2-mm notch was made, and the end of the glass capillary was sealed prior to each experiment using a VersaLaser™ [24]. This allowed for efficient kinetic mixing before the exchange reaction was quenched by the acid and sent to the PMMA chip containing the pepsin agarose beads for MS1 fragmentation. The duration of the D_2_O–protein exchange reaction is controlled by varying the length of the capillary past the protein–D_2_O mixing point. In this study, 5-mm and 10-mm long capillaries were used post-mixing, corresponding to 2.07 s (approximately 2 s) and 4.14 s (approximately 4 s), respectively.

Data was collected in IMS mode in the 400–1500 *m/z* range. The experimental deuterium uptake of each peptide obtained was calculated using a custom-built software program (DJW, unpublished results). 

Each set of data was collected on the same day, including six sets of 3 × 5 min spectra acquisitions of protein GSNOR without deuterium exchange, 2-s exchange, and 4-s exchange. This was followed by two sets of 3 × 5 min spectra acquisitions of protein GSNOR in the presence of a 20-fold molar excess of GSNO with deuterium exchange: 2-s exchange, and 4-s exchange. Concentrations of GSNOR and GSNO were calculated by Bradford assay and the GSNO extinction coefficient (λ_max_ = 335 nm, ε_M_ = 920 M^−1^ cm^−1^), respectively, to confirm a 20× stoichiometric addition of GSNO. Integrity of the notch was confirmed to be maintained at the end of the experiment.

## 3. Results

### 3.1. GSNOR Steady-State Kinetics Display Allosteric Behavior 

**Figure 1:**
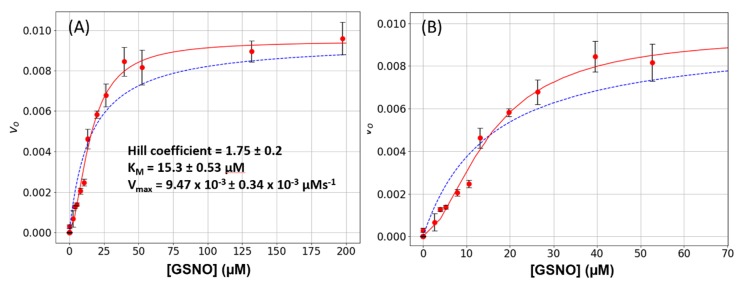
*S*-nitrosoglutathione reductase (GSNOR) steady-state kinetics. (**A**) Varying amounts of S-nitrosoglutathione (GSNO) (0 to 200 μM) plus a constant amount of NADH (80 μM) were added to a 1-mL cuvette along with 400 μL of phosphate-buffered saline (PBS). The absorbance was monitored for 15 s to establish the blank rate at which time a constant volume of purified recombinant GSNOR (5 μL corresponding to a final concentration of 20 nM) was rapidly added to the assay mixture with the aid of a plumper. The change in absorbance was monitored for a further 60 s. The net enzymatic initial rates were calculated for each GSNO (red circles). The steady-state kinetic parameters were estimated from a fit of the data to the Michaelis–Menten (dashed blue line) or the Hill–Langmuir (red line) algorithms. The error bars represent SD, *n* = 4. (**B**) The same data as in Figure 1A displayed with a narrower GSNO range to emphasize the sigmoidal behavior of the data.

Wild-type GSNOR was subjected to a steady-state kinetic study to estimate its Michaelis constants K_M_ and V_max_ for GSNO. These experiments were performed as a function of varying GSNO (2 μM to 200 μM) with the cofactor NADH, being held constant at 80 μM. The plots of [GSNO] versus initial rates, *v_o_*, displayed sigmoidal characteristics, and the data could not be fitted to a simple hyperbola (Figure 1A,B, dashed blue lines). However, the data was well accommodated by the Hill–Langmuir equation (Equation (1)) with an estimated Hill coefficient of 1.75 ± 0.20, a K_M_ of 15.3 ± 0.53 μM, and a V_max_ of 9.47 × 10^−3^ ± 0.34 × 10^−3^ Μm s^−1^ (Figure 1A,B, red lines). These experiments were repeated 8-times on different days with 3 different batches of enzyme. A positive Hill coefficient is ascribed to positive cooperativity. Since the only variable in these experiments is GSNO, we hypothesize that as its concentration increases, GSNO binds to an allosteric site that is distinct from the active site and converts the enzyme to a more active conformation. This phenomenon is clearly observable here (Figure 1B): the initial rates increase linearly from approximately 2 to 12 μM, above which there is an inflection point and the rates increase with a larger slope. 

In order to test this hypothesis, molecular dynamics (MD) simulations were initiated to search for a putative GSNO binding site on GSNOR.

### 3.2. Docking and MD Simulations Identify a Putative GSNO Binding Site on GSNOR

**Figure 2:**
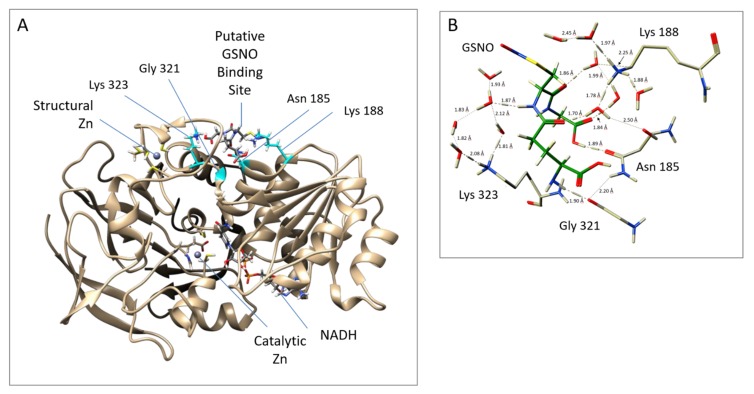
Putative allosteric site near the structural zinc, as obtained from MD simulations. (**A**) GSNOR A Chain (template structure– PDB ID: 3QJ5 [21]) with GSNO bound to Asn_185_ and Gly_321_ Lys_188_ and Lys_323_; (**B**) Close up of the interactions between GSNOR residues and GSNO. The protein structure was visualized with a UCSF Chimera 1.11.2.

Notably, the docking and MD studies implicated four amino acids in the binding of GSNO at a putative allosteric site. The putative allosteric GSNO-binding domain and the implicated amino acid residues are displayed in Figure 2A,B, respectively. More specifically, the results suggest that GSNO can hydrogen bond directly with Asn_185_ and Gly_321_. Concomitantly, GSNO interacts with Lys188 and Lys323 via a solvent network of hydrogen bonds (see Figure 2B). Docking studies were performed on GSNO binding both within the active site and the putative allosteric site. The docking scores of the 10 best (preferred) binding modes of GSNO in each site are given in Table S1. The scores obtained, while not quantitative, represent the binding affinity; that is, lower scores indicate more favourable and stable interactions. Notably, the top-ranked docked active site bound–GSNO complex had a score of −8.60 kcal mol^−1^, while the top-ranked docked GSNO–putative allosteric site gave a comparable score of −10.4 kcal mol^−1^ [25].

### 3.3. HDX-MS Initiated to Probe for the Postulated GSNO-Binding Site

To do this, we used a short-labeling time HDX, which is a technique that is uniquely sensitive (compared to conventional HDX) to weak ligand binding and subtle shifts in conformational dynamics [26]. Nine sets of GSNOR D-incorporation data were successfully analyzed (Table S2). With the ‘bottom–up’ workflow being employed here, the electrospray ionization (ESI) mass spectra recorded are of a mixture of peptides resulting from the digestion of GSNO at pH 2.4 (where the HDX labeling reaction is quenched). Sample ‘baseline’ (no deuterium) ESI spectra for selected peptides, with peak distributions arising only from heavy-atom isotopes (i.e., ^13^C, ^15^N, and ^18^O), are shown in Figure S1. As deuterium is incorporated (Figure S1), the peak distribution shifts with the addition of the heavier isotope. The new distribution can then be deconvoluted to yield the percentage occupancy of backbone amide sites by deuterium (usually given as % uptake). In comparative HDX analyses, the percentage uptake parameter is compared across multiple labeling times for two states of the protein (i.e., ligand-free versus ligand-bound). This work generally follows the data collection guidelines and reports suggestions outlined in a recent report [27] with some exceptions due to the unique rapid microfluidic setup used. In differential labeling experiments, short time-scale (2 s and 4 s) HDX measurements provide extra sensitivity to subtle changes in conformational dynamics often associated with allostery, while still allowing the detection of less subtle changes that are a direct result of ligand binding. In this case, the differential measurements were for free GSNOR (~1 µM) and GSNOR (~1 μM) in the presence of a 20-fold molar excess of GSNO.

The complete set of HDX-MS data are reported in the Supplementary Materials section (Table S3). 

**Figure 3:**
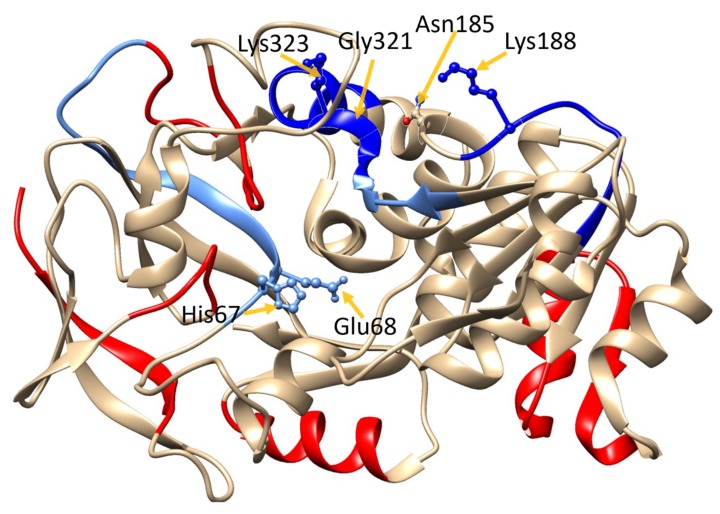
Hydrogen–deuterium exchange (HDX)-MS heat map of GSNOR. GSNOR crystal structure (PDB ID: 3QJ5 [21]) with D-uptake superimposed color-code based on Δ4s + Δ2s data (column 11 Table S2) data: **red: >Δ1%**; **cyan: 0% to Δ –0.5%**; **sky blue: Δ –0.5% to Δ −1%**; **blue: >−1%.** The protein structure was visualized with UCSF Chimera 1.11.2.

Four amino acid residues were implicated by the MD studies of these peptides containing Lys_188_; Gly_321_ and Lys_323_ were detected in the MS/HDX experiments with Gly_321_, and Lys_323_ appeared in two overlapping peptide fragments. Invariably, these peptides showed a decreased uptake in D-uptake in the presence of GSNO (Lys_188_: −1.78%; Gly_321_ and Lys_323_: −1.00% to −1.61%), strongly supporting the identification of this region as a binding site for GSNO (Table S3, Figure 3). MS/HDX data could not be obtained for Asn_185_ (although adjacent peptides exhibited a significantly decreased uptake). 

In addition, there was a statistically significant decrease (−0.83 ± 0.08) in d-uptake in the presence of GSNO, in a peptide containing the catalytic site residues His_67_ and Glu_68_ (GHEGAGIVESVGEGVT). Observing this decrease in dynamics around the active site is expected since the substrate, GSNO, is bound to the active site but not turned over during the HDX experiments (Table S2). The fact that GSNO-induced decrease is also observed at the catalytic site further validates the HDX experiments performed here.

The HDX-MS data also indicates three hot peptides (red, with > 1% d-uptake) in the 2-s data that increase to 8 in the 4-s data. In the combined 2s + 4s data, these hot regions increase to 10 peptides. One explanation for these hot regions is that GSNO binding to the allosteric site ‘primes’ the enzyme for catalysis by lowering one or more activation barriers for conformational transitions associated with catalysis, as previously observed with chymotrypsin [28]. 

### 3.4. Site-Directed Mutation of Lys Residues at the Putative GSNO Allosteric Site Eliminates Allosteric Behavior

**Figure 4:**
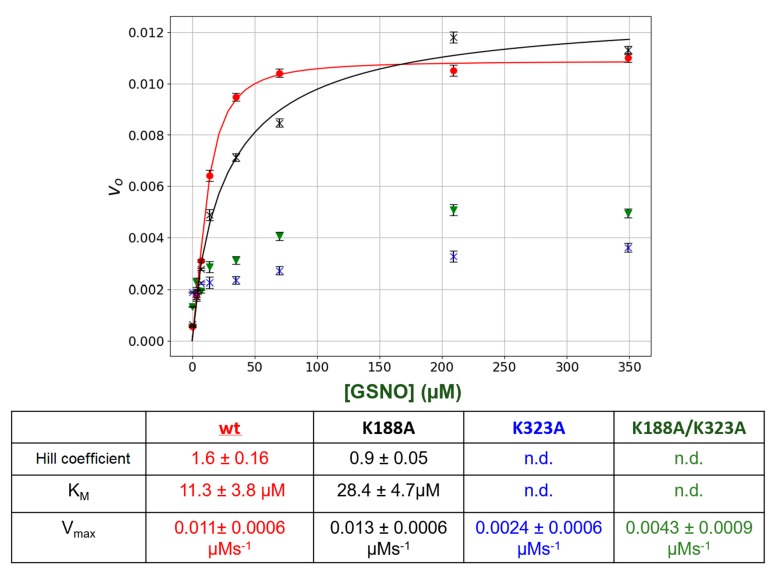
Steady-state kinetics of GSNOR *wt* and mutants. Varying amounts of GSNO (0 to 200 μM) plus a constant amount of NADH (80 μM) were added to a 1-mL cuvette along with 400 μL of phosphate-buffered saline (PBS). The absorbance was monitored for 15 s to establish the blank rate at which time a constant volume of purified recombinant GSNOR (*wt* or mutants) (5 μL corresponding to a final concentration of 20 nm) was rapidly added to the assay mixture with the aid of a plumper. The change in absorbance was monitored for a further 60 s. The net enzymatic initial rates were calculated for each GSNO. ***Wt *(●)**; **K188A (X)**; **K323A (▼); K188A/K323A (X).** The steady-state kinetic parameters were estimated from a fit of the data (red or black lines) to the Hill–Langmuir algorithm. The error bars represent SD, *n* = 4. The table below the figure summarizes the kinetic parameters extracted from the data in the figure.

The lysine residues in the putative GSNO-binding site, Lys _188_ and Lys_323_, were singly or doubly mutated. Then, the kinetic behavior of the mutant enzymes was compared to *wt* GSNOR. The results are summarized in Figure 4. Mutation of the Lys_188_ to an Ala (K188A) resulted the loss of allosteric behavior as initial rates versus [GSNO] plots no longer displayed sigmoidal behavior (Figure 4), as the Hill coefficient was 0.9 ± 0.05 as opposed to that of 1.6 ± 0.16 for the *wt* GSNOR. In addition, the estimated K_M_ increased by approximately 2.6-fold. The mutation of the Lys_323_ (K323A) drastically lowered the initial rates resulting in an estimated V_max_ that was approximately 80% less than that of the *wt* GSNOR. Similarly, the double mutants (K188A/K323A) also yielded ~60% lower V_max_ in comparison to the *wt* enzyme. The K_M_ as well as the Hill coefficient could not be estimated for both K323A and the double mutant, K188A/K323A, with confidence due to the low initial rates.

## 4. Discussion

The postulated physiological role of GSNOR is to irreversibly remove nitric oxide equivalents, thereby attenuating protein *S*-nitrosylation and blocking SNO-signaling pathways. 

In this study, we observed that under steady-state kinetic conditions, the catalysis of GSNO reduction by GSNOR did not follow simple hyperbolic behavior i.e., the initial rate versus GSNO plots were sigmoidal (Figure 1). These data were well accommodated by the Hill–Langmuir algorithm for allosteric kinetic behavior (Equation (3)) with an estimate Hill coefficient of approximately 1.5, which indicates positive cooperativity; that is, as the GSNO concentrations increase, the enzyme becomes activated. To the best of our knowledge, GSNOR allostery has not been reported previously. Allostery is manifested in multimeric proteins such as hemoglobin where allosteric behavior was first observed and characterized. In hemoglobin, as O_2_ tension increases, one of its four subunits undergoes a conformational change that is translated to the other subunits, lowering their affinity to O_2_. GSNOR exists as a homodimer; therefore, it possesses the minimum structural requirement for allosteric behavior. The kinetic behavior observed here with GSNOR can be explained by its substrate GSNO binding to an allosteric site on the enzyme and changing its conformation of its subunit(s) to activate the enzyme at larger levels of GSNO. The substrate-induced activation of GSNOR is logical from the physiological point of view, as GSNOR would work more efficiently to remove GSNO upon the sensing of a larger GSNO-flux.

We next probed the GSNOR structure for potential GSNO allosteric domains via dynamics and docking studies utilizing the GSNOR crystal structure PDB ID: 3QJ5 [21] as the template structure. The computational investigations revealed a stable GSNO complex with a tetrad of residues Asn_185_, Lys_188_, Gly_321_, and Lys_323_ (Figure 2). In fact, this postulated GSNO allosteric site yielded a more stable GSNO domain complex in comparison to that observed for the active site GSNO-binding domain (Table S1).

The existence of the GSNO allosteric domain was tested with HDX-MS. In these experiments, D-exchange was performed on the protein in the absence and presence of GSNO. In theory, if GSNO bound at or near the residues comprising the GSNO-binding domain, then these residues could be identified as they would have less exposure to solvent and as a result yield lower D-exchange endpoints. The HDX-MS was performed at two exchange endpoints, 2 s and 4 s. The 2-s data corroborated the dynamics and docking studies as the postulated GSNO allosteric site residues Lys_188_, Gly_321_, and Lys_323_, as well as the active site residues, showed decreased D-exchange endpoints (Table S2, Figure 3). At the 4-s D-exchange, the postulated allosteric site residue Lys_188_ decreased further, whereas Gly_321_ and Lys_323_ as well as the active site residues yielded increased D-exchange endpoints, indicating more solvent exposure. We interpret these data as evidence that GSNO binding to the allosteric site ‘primes’ the enzyme for catalysis by lowering one or more activation barriers for the conformational transitions associated with catalysis.

As further confirmatory evidence for the GSNO allosteric site, we employed site-directed mutagenesis to change the postulated allosteric site lysines to alanines (K188A and K323A). Interestingly, K188A totally eliminated the +ve allosteric kinetic behavior as the intial rate (*v_o_*) versus GSNO plots no longer displayed sigmoidal behavior (Figure 4). The alteration of the other lysine (K323A) drastically lowered the catalytic activity of the enzyme, once again indicating that any changes in this region of the protein can drastically affect GSNOR structure and function. Double mutation of the lysines (K188A/K323A) again essentially inactivated the enzyme.

The HXD and the mutagenesis studies strongly suggest that GSNOR contains a second allosteric binding site for GSNOR. 

## 5. Conclusions

We have for the first time detected allosteric behavior in GSNOR and with the integration of molecular dynamics/docking studies, HDX-MS and site-directed mutagenesis identified a putative allosteric GSNO-binding domain. Furthermore, these studies have demonstrated that the activity of GSNOR is very sensitive to structural perturbations at this domain, which is remote from the active site. Clearly, controlling the activity of GSNOR would be of great pharmacological interest. The results of our current study could potentially provide this opportunity as this apparently hypersensitive domain can be targeted by small molecules to attenuate GSNOR activity.

## Supplementary Materials

The following are available online at https://www.mdpi.com/2076-3921/8/11/545/s1, Figure S1: Representative peptide maps to visualize d-uptake, Table S1: Estimated binding energies (kcal mol^−1^) obtained from docking studies of GSNO in the putative allosteric site and active site of GSNOR, Table S2: Full peptide list resulting from MS-MS identification, Table S3: 2 s and 4 s d-Uptake data in the presence and absence of GSNO
