# Supplementary material for: Evidence for an Allosteric S-Nitrosoglutathione Binding Site in S-Nitrosoglutathione Reductase (GSNOR)
Source: Antioxidants (Basel). 2019 Nov 13;8(11):545. doi: 10.3390/antiox8110545 (PMC6928738; doi:10.3390/antiox8110545)
Supplement: Supplementary file 1 [file antioxidants-08-00545-s001.pdf]

Table S1. Estimated binding energies (kcal mol^{-1}) obtained from docking studies of GSNO in the putative allosteric site and active site of GSNOR.

	**Allosteric Site**	**Active Site**
	**Binding Energy**	**Binding Energy**
**1**	-10.45	-8.60
**2**	-9.69	-8.23
**3**	-9.30	-8.08
**4**	-8.47	-8.05
**5**	-8.36	-7.99
**6**	-8.19	-7.98
**7**	-8.14	-7.89
**8**	-8.07	-7.88
**9**	-7.89	-7.87
**10**	-7.81	-7.86

Table S2. Full peptide list resulting from MS-MS identification.

m/z	Theoretical Amino Acid Number	Experimental Amino Acid Number	Amino Acid Sequence
813.3543	0-6	20-26	(S)HMANEVI(K)
690.0621	7-11	27-33	(I)KCKAAVA/(W)
621.4647	11-22	31-42	(A)/AVAWEAGKPLSI(E)
545.9043	25-43	45-54	(E)/IEVAPPKAHE/(V)
406.9651	35-41	55-61	(E)/VRIKIIA/(T)
457.5103	35-42	55-62	(E)/VRIKIIAT(A)
508.7969	42-56	62-76	(A)/TAVCHTDAYTLSGAD(P)
462.4796	52-56	72-76	(T)LSGAD(P)
762.2355	64-79	84-99	(V)ILGHEGAGIVESVGEG(V)
749.1952	66-81	86-101	(L)/GHEGAGIVESVGEGVT(K)
1260.826	75-87	95-107	(E)/SVGEGVTKLKAGD(T)
1017.545	78-87	98-107	(G)EGVTKLKAGD(T)
889.3751	91-98	111-118	(I)PLYIPQCG(E)
863.8682	131-138	151-158	(F)/TCKGKTIL/(H)
796.6921	139-145	159-165	(L)/HYMGTST(F)
903.3278	187-195	207-215	(T)AKLEPGSVC(A)
646.5185	190-203	210-223	(L)/EPGSVCAVFGLGGV(G)
1091.721	194-205	214-225	(S)VCAVFGLGGVGL/(A)
1074.582	210-220	230-240	(M)GCKVAGASRII(G)
537.7998	211-221	231-241	(G)CKVAGASRIIG(V)
950.457	223-230	243-250	(V)DINKDKFA/(R)
1053.654	228-236	248-256	(D)KFARAKEFG(A)
669.6493	231-242	251-262	(A)/RAKEFGATECIN(P)
726.2026	233-246	253-265	(A)/KEFGATECINPQD(F)
818.1564	239-245	259-265	(T)ECINPQD(F)
1061.054	246-254	266-274	(D)FSKPIQEVL/(I)
1038.94	315-324	335-344	(W)/KGTAFGGWKS(V)
1173.876	320-330	340-350	(F)/GGWKSVESVPK(L)
587.3912	324-334	344-354	(K)SVESVPKLVSE/(Y)
1241.154	334-343	354-363	(S)EYMSKKIKVD(E)
965.4494	353-360	373-380	(F)/DEINKAFE/(L)
1231.957	354-363	374-383	(D)EINKAFELMH(S)

Theoretical amino acid number corresponds to labels beginning at Met1, whereas experimental amino acid numbers include the His tags of the recombinant protein. 292 total amino acids have been sequenced, however only 188 unique amino acids have coverage, resulting in a 52% sequence coverage of the 374 relevant residues.

Table B.2: Representative peptide to visualize deuterium uptake.

Peak information: 889.6 m/z, and the amino acid sequence is (I)PLYIPQCG(E) of residues 91-98.

Deuterium uptake (D) and change of deuterium uptake (ΔD) is displayed.

**GSNOR HDX-MS:**		**GSNOR + GSNO HDX-MS:**	
No Deuterium Exchange	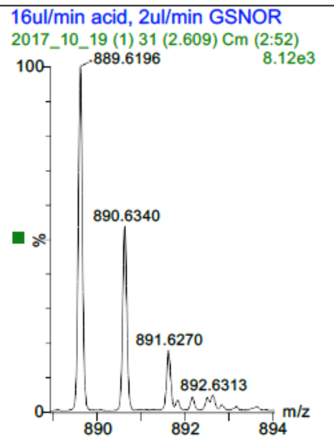	No Deuterium Exchange	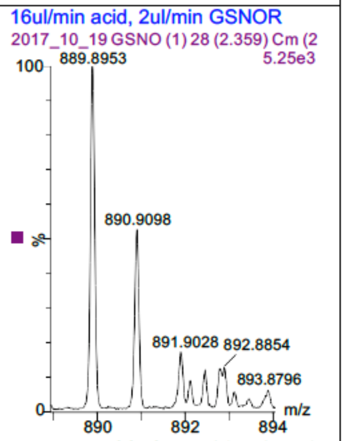
Two sec Exchange D: 24.4% ± 2.1	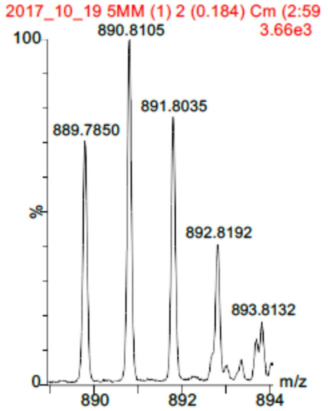	Two sec Exchange D: 24.0% ± 0.3<br><br>ΔD: -0.4%	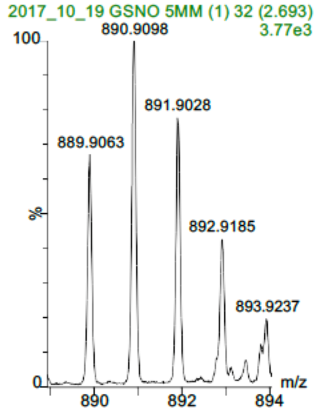
Four sec Exchange D: 27.1% ± 1.6	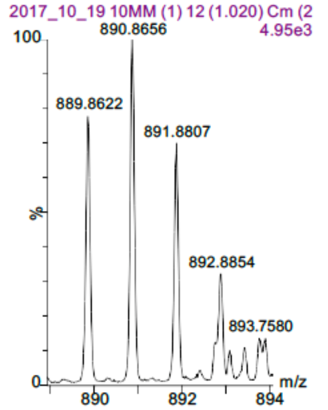	Four sec Exchange D: 27.5% ± 0.5<br><br>ΔD: +0.4%	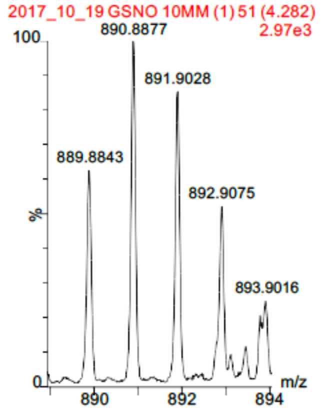

Figure S1. Representative peptide maps to visualize D-uptake.

Table S3. 2s and 4s D-Uptake data in the presence and absence of GSNO.

Baseline D-Uptake (%) (n=6)	Std. Dev.	[2s] +GSNO D-Uptake (%) (n=2)	Std. Dev.	[2s] ΔD-Uptake	Std. Dev.	[4s] +GSNO D-Uptake (%) (n=2)	Std. Dev.	[4s] ΔD-Uptake	Std. Dev.	Δ4s+Δ2s	Std. Dev.	Peptide Sequence Range	Peptide Sequence
17.20	1.42	17.00	1.60	-0.20	0.03	18.67	0.60	1.47	0.06	1.27	0.05	0-6	(S)HMANEVI(K)
15.89	2.75	15.83	1.65	-0.06	0.01	17.67	0.94	1.78	1.00	1.72	0.51	7-13	(I)KCKAAVA/(W)
12.67	1.12	12.83	1.65	0.17	0.02	13.17	0.24	0.50	0.28	0.67	0.15	11-22	(A)/AVAWEAGKPLSI(E)
17.00	1.15	16.67	0.94	-0.33	0.02	17.67	0.00	0.67	0.37	0.33	0.19	25-34	(E)/IEVAPPKAHE/(V)
21.56	1.52	20.17	0.71	-1.39	0.07	21.17	0.71	-0.39	0.22	-1.78	0.15	35-42	(E)/VRIKIIAT(A)
11.94	1.15	11.50	0.71	-0.44	0.04	12.17	0.24	0.22	0.13	-0.22	0.08	42-56	(A)/TAVCHTDAYTLSGAD(P)
20.33	2.08	19.17	0.71	-1.17	0.08	21.50	0.71	1.17	0.64	0.00	0.36	52-56	(T)LSGAD(P)
9.50	0.88	9.17	0.24	-0.33	0.02	10.00	0.00	0.50	0.27	0.17	0.14	64-79	(V)ILGHEGAGIVESVGEG(V)
9.67	0.67	9.00	1.41	-0.67	0.07	9.50	0.71	-0.17	0.09	-0.83	0.08	66-81	(L)/GHEGAGIVESVGEGVT(K)
18.40	2.45	20.83	1.65	2.43	0.25	23.50	4.95	5.10	2.60	7.53	1.43	78-87	(G)EGVTKLKAGD(T)
24.44	2.13	24.00	0.47	-0.44	0.02	27.50	0.71	3.06	1.65	2.61	0.84	91-98	(I)PLYIPQCG(E)
15.67	1.35	15.33	0.60	-0.33	0.02	16.00	0.55	0.33	0.19	0.00	0.10	131-138	(F)/TCKGKTIL/(H)
16.75	1.66	17.00	0.00	0.25	0.01	17.67	0.50	0.92	0.46	1.17	0.24	139-145	(L)/HYMGTST(F)
25.72	2.78	25.33	1.20	-0.39	0.03	24.33	0.50	-1.39	0.85	-1.78	0.44	187-195	**(T)AKLEPGSVC(A)**
19.25	1.64	18.33	1.41	-0.92	0.07	18.50	2.59	-0.75	0.43	-1.67	0.25	190-203	(L)/EPGSVCAVFGLGGV(G)
9.28	0.68	9.00	0.47	-0.28	0.02	9.67	0.94	0.39	0.21	0.11	0.12	184-205	(S)VCAVFGLGGVGL/(A)
16.83	2.50	15.00	0.50	-1.83	0.17	13.67	0.60	-3.17	2.09	-5.00	1.13	210-220	(M)GCKVAGASRII(G)
15.13	1.24	15.33	0.60	0.20	0.01	16.33	0.47	1.20	0.65	1.40	0.33	223-230	(V)DINKDKFA/(R)
13.39	1.43	12.67	0.94	-0.72	0.07	13.17	0.24	-0.22	0.13	-0.94	0.10	228-236	(D)KFARAKEFG(A)
13.83	0.50	15.67	0.60	1.83	0.07	14.67	0.60	0.83	0.47	2.67	0.27	231-242	(A)/RAKEFGATECIN(P)
19.89	2.30	23.33	0.60	3.44	0.23	23.67	0.60	3.78	2.09	7.22	1.16	233-245	(A)/KEFGATECINPQD(F)
29.53	3.46	29.67		0.13	0.02	30.67	0.60	1.13	0.61	1.27	0.31	239-245	(T)ECINPQD(F)
17.07	1.76	15.83	1.65	-1.23	0.13	12.17	5.42	-4.90	3.24	-6.13	1.68	246-254	(D)FSKPIQEVL/(I)
15.92	2.45	15.00	1.41	-0.92	0.11	15.83	0.24	-0.08	0.05	-1.00	0.08	315-324	**(W)/KGTAFGGWKS(V)**
13.72	1.33	12.33	0.47	-1.39	0.10	13.50	0.24	-0.22	0.13	-1.61	0.11	320-330	**(F)/GGWKSVESVPK(L)**
14.78	1.34	14.33	0.47	-0.44	0.03	15.33	0.47	0.56	0.31	0.11	0.17	324-334	(K)SVESVPKLVSE/(Y)
15.00	1.14	15.33	0.60	0.33	0.02	15.33	0.50	0.33	0.19	0.67	0.10	334-343	(S)EYMSKKIKVD(E)
19.33	1.33	19.00	0.94	-0.33	0.02	19.67	0.47	0.33	0.19	0.00	0.10	353-360	(F)/DEINKAFE/(L)
17.61	1.38	18.17	0.24	0.56	0.03	18.17	0.71	0.56	0.31	1.11	0.17	354-363	(D)EINKAFELMH(S)

The 1st column in Table S2 is the baseline D-uptake (%) for GSNOR peptides in the absence of GSNO. The 3rd and 7th columns are D-uptake (%) in the presence of GSNO for 2 s or 4 s, respectively. The 5th and 9th columns are the difference (Δ) in D-uptake in the presence of GSNO for 2 s or 4 s (column 3 minus column 1 = 2s ΔD-uptake; column 7minus column 1 = 4s ΔD-uptake). In column 11, 2s ΔD-uptake and 4s ΔD-uptake are added together to get a more accurate picture of the global ligand-induced conformational changes on GSNOR.
